# High-Intensity Acceleration and Deceleration Demands in Elite Team Sports Competitive Match Play: A Systematic Review and Meta-Analysis of Observational Studies

**DOI:** 10.1007/s40279-019-01170-1

**Published:** 2019-09-10

**Authors:** Damian J. Harper, Christopher Carling, John Kiely

**Affiliations:** 1 School of Sport, York St John University, Lord Mayors Walk, York, YO31 7EX UK; 2 Institute of Coaching and Performance, School of Sport and Wellbeing, University of Central Lancashire, Preston, PR1 2HE UK

## Abstract

**Background:**

The external movement loads imposed on players during competitive team sports are commonly measured using global positioning system devices. Information gleaned from analyses is employed to calibrate physical conditioning and injury prevention strategies with the external loads imposed during match play. Intense accelerations and decelerations are considered particularly important indicators of external load. However, to date, no prior meta-analysis has compared high and very high intensity acceleration and deceleration demands in elite team sports during competitive match play.

**Objective:**

The objective of this systematic review and meta-analysis was to quantify and compare high and very high intensity acceleration vs. deceleration demands occurring during competitive match play in elite team sport contexts.

**Methods:**

A systematic review of four electronic databases (CINAHL, MEDLINE, SPORTDiscus, Web of Science) was conducted to identify peer-reviewed articles published between January 2010 and April 2018 that had reported higher intensity (> 2.5 m·s^−2^) accelerations and decelerations concurrently in elite team sports competitive match play. A Boolean search phrase was developed using key words synonymous to team sports (population), acceleration and deceleration (comparators) and match play (outcome). Articles only eligible for meta-analysis were those that reported either or both high (> 2.5 m·s^−2^) and very high (> 3.5 m·s^−2^) intensity accelerations and decelerations concurrently using global positioning system devices (sampling rate: ≥ 5 Hz) during elite able-bodied (mean age: ≥ 18 years) team sports competitive match play (match time: ≥ 75%). Separate inverse random-effects meta-analyses were conducted to compare: (1) standardised mean differences (SMDs) in the frequency of high and very high intensity accelerations and decelerations occurring during match play, and (2) SMDs of temporal changes in high and very high intensity accelerations and decelerations across first and second half periods of match play. Using recent guidelines recommended for the collection, processing and reporting of global positioning system data, a checklist was produced to help inform a judgement about the methodological limitations (risk of detection bias) aligned to ‘data collection’, ‘data processing’ and ‘normative profile’ for each eligible study. For each study, each outcome was rated as either ‘low’, ‘unclear’ or ‘high’ risk of bias.

**Results:**

A total of 19 studies met the eligibility criteria, comprising seven team sports including American Football (*n *= 1), Australian Football (*n* = 2), hockey (*n* = 1), rugby league (*n* = 4), rugby sevens (*n* = 3), rugby union (*n* = 2) and soccer (*n* = 6) with a total of 469 male participants (mean age: 18–29 years). Analysis showed only American Football reported a greater frequency of high (SMD = 1.26; 95% confidence interval [CI] 1.06–1.43) and very high (SMD = 0.19; 95% CI − 0.42 to 0.80) intensity accelerations compared to decelerations. All other sports had a greater frequency of high and very high intensity decelerations compared to accelerations, with soccer demonstrating the greatest difference for both the high (SMD = − 1.74; 95% CI − 1.28 to − 2.21) and very high (SMD = − 3.19; 95% CI − 2.05 to − 4.33) intensity categories. When examining the temporal changes from the first to the second half periods of match play, there was a small decrease in both the frequency of high and very high intensity accelerations (SMD = 0.50 and 0.49, respectively) and decelerations (SMD = 0.42 and 0.46, respectively). The greatest risk of bias (40% ‘high’ risk of bias) observed across studies was in the ‘data collection’ procedures. The lowest risk of bias (35% ‘low’ risk of bias) was found in the development of a ‘normative profile’.

**Conclusions:**

To ensure that elite players are optimally prepared for the high-intensity accelerations and decelerations imposed during competitive match play, it is imperative that players are exposed to comparable demands under controlled training conditions. The results of this meta-analysis, accordingly, can inform practical training designs. Finally, guidelines and recommendations for conducting future research, using global positioning system devices, are suggested.

**Electronic supplementary material:**

The online version of this article (10.1007/s40279-019-01170-1) contains supplementary material, which is available to authorized users.

## Key Points

**Table:** 

All team sports apart from American Football reported a greater frequency of high and very high intensity decelerations compared to accelerations. Importantly, the damaging consequences of frequent and intense decelerations imply that specific loading strategies, to inoculate players from negative deceleration outcomes, may be advisable.
There was a small decrease in the frequency of high and very high intensity accelerations and decelerations from the first to the second half periods of elite competitive match play, suggesting intense accelerations and decelerations could be particularly vulnerable to neuromuscular fatigue and consequently to an exacerbated risk of incurring injury.
In advancing the specificity of acceleration and deceleration training prescriptions, future research should look to ‘individualise’ and ‘contextualise’ acceleration and deceleration occurrences during match play.

## Introduction

Team sports competitive match play requires players to perform frequent intense acceleration and deceleration actions. At the highest standard of competitive match play, there has been an evolutionary progression in the high-intensity work load profile of the contemporary team sports player [1–4]. Intense accelerations and decelerations make up a substantial part of the high-intensity external workload, yet impose distinctive and disparate internal physiological and mechanical loading demands on players [5]. For example, accelerations have a higher metabolic cost [6], whereas decelerations have a higher mechanical load [7] likely caused by high-force impact peaks and loading rates [8] that can inflict greater damage on soft-tissue structures especially if these high forces cannot be attenuated efficiently [9]. As such, the frequency of high-intensity accelerations and decelerations completed during match play is commonly associated with decrements in neuromuscular performance capacity and indicators of muscle damage post-match [10–13]. Despite these effects, elite athletes are more capable of maintaining a higher frequency and magnitude of accelerations and decelerations than lower performing players, which can contribute to enhanced match play performance outcomes that require rapid changes in velocity to be made [14, 15].

Research has also shown that during elite team sports competitive match play there is a second half decline in the frequency and distance spent accelerating and decelerating at high intensity [16–20], suggesting that these actions may be particularly sensitive to fatigue development and injury risk [21]. Collation and analysis of data from studies reporting temporal changes in the occurrence of higher intensity accelerations and decelerations during competitive match play would help acquire knowledge regarding the magnitude of the decline and potential impact that this may have on match performance and injury risk. Therefore, careful monitoring of each of these specific actions during training and match play is of significant importance to effective player load management systems, and is common practice amongst practitioners working with players at the elite level [22].

Global positioning system (GPS) devices are most commonly used to quantify the occurrence and characteristics of higher intensity accelerations and decelerations during competitive match play. With rapid advancements in this technology, together with approval by sports governing bodies to allow usage within official competitive match play, there has been an exponential increase in studies that have reported data on match-play movement demands. The results of this research have been summarised in recent systematic reviews and meta-analyses [23–25]. Despite this knowledge base, there is currently no systematic review or meta-analysis that has specifically focused on quantifying and comparing the occurrence of higher intensity accelerations and decelerations during competitive match play across a range of team sports in elite players. Furthermore, given the evident importance of these actions and the increasing number of studies measuring these actions using GPS devices there is also a need to systematically appraise the methodological procedures being used with view to identifying potential or necessary improvements in current practice.

Therefore, the aim of this systematic review and meta-analysis was to compare high (> 2.5 m·s^−2^) and very high (> 3.5 m·s^−2^) intensity acceleration and deceleration demands in elite team sports competitive match play. A temporal analysis of changes in the frequency of high and very high intensity accelerations and decelerations from the first to the second half periods of match play was also performed. A secondary aim was to review the methodological procedures used to quantify the occurrence of high and very high intensity accelerations and decelerations during elite competitive match play when measured using GPS devices.

## Methods

### Study Design

The planning and documentation of the current review were conducted in accordance with PRISMA (Preferred Reporting Items for Systematic Review and Meta-analysis) guidelines [26], with a meta-analysis following the Cochrane collaboration guidelines [27].

### Search Strategy

Systematic searches of four electronic databases (CINAHL, MEDLINE, SPORTDiscus, Web of Science) were conducted by the lead author (DH) to identify peer-reviewed articles published in the English language between 1 January, 2010 and 1 April, 2018. The search strategy was developed using PICO (population, intervention, comparator, outcome) elements [26]. Related search terms synonymous to team sports (population), acceleration and deceleration (comparators), and match play (outcomes) were developed in accordance with those used by McLaren et al. [28]. Additional search terms were identified from pilot searching (screening of titles, abstracts and full text of papers previously known). Boolean operators ‘OR’ and ‘AND’ were used to construct the final search phrase (Table 1).

**Table 1:** Database search strategy

Key search terms	Related search terms
1. Acceleration/deceleration	accelerat* **OR** decelerat* **OR** GPS **OR** “global positioning system*” **OR** “GPS output*” **OR** microtechnology **OR “**micromechanical-electrical system*” **OR** microsensor* **OR** “tracking system*” **OR** video* **OR** camera* **OR** “time-motion” **OR** “match analysis system” **OR** “notational analysis” **OR** “multi-camera system” **OR** “external load*” **OR** “external training load*” **OR** “external intensit*” **OR “**external work” **OR** workload* **OR** “physical performance*” **OR** “physical demand*” **OR** “physical exertion” **OR** acceleromet* **OR** “inertial measurement unit” **OR** activit* **OR** “activity analysis” **OR** “activity demand” **OR** “activity profile* **OR** “movement analysis” **OR “**movement performance*” **OR** “movement demand*” **OR** “movement pattern*” **OR “**movement profile*” **OR** velocit* **OR** “high-velocit*” **OR** speed* **OR** “high-speed*” **OR** “maximal-speed” OR “running intensit*” **OR** “high-intensit*” **OR** energ* **OR** “energy cost*” **OR** “accelerometer load*” **OR** “body load*” **OR** “Player Load*” **OR** “PlayerLoad*” **OR** “metabolic power” **OR** “metabolic load” **OR** “high power distance”
2. Team-sport	team-sport* **OR** “multi-direction*” **OR “**field sport*” **OR** “field-based sport*” **OR “**intermittent sport*” **OR** soccer **OR** ‘‘soccer player*’’ **OR** footballer* **OR** ‘‘football player*’’ **OR** futsal **OR** ‘‘futsal player*’’ **OR** rugby **OR** ‘‘rugby football*’’ **OR** ‘‘rugby player*’’ **OR** ‘‘rugby football player*’’ **OR** ‘‘rugby union’’ **OR** ‘‘rugby union player*’’ **OR** ‘‘rugby league’’ **OR** ‘‘rugby league player*’’ **OR “**rugby sevens” **OR** “rugby sevens player” **OR “**American football*” **OR “**American football player” **OR “**national collegiate athletic association*” **OR** NCAA **OR** ‘‘Australian rules football*’’ **OR** ‘‘Australian football*’’ **OR** ‘‘Australian rules football player*’’ **OR** ‘‘Australian football player*’’ **OR** AFL **OR** ‘‘Gaelic football*’’ **OR** ‘‘Gaelic football player*’’ **OR** hurling **OR** ‘‘hurling player*’’ **OR** hurler* **OR** basketball **OR** basketballer* **OR** ‘‘basketball player*’’ **OR** handball* **OR** ‘‘handball player*’’ **OR** handballer* **OR** hockey **OR** ‘‘hockey player*’’ **OR** lacrosse **OR** ‘‘lacrosse player*’’ **OR** netball **OR** ‘‘netball player*’’ **OR** netballer*
3. Match-play	match-play* **OR** “match activit*” **OR “**match analysis” **OR** “match performance*” **OR “**match demand*” **OR** “match running” **OR “**match intensit*” **OR** “match event*” **OR** “match profile*” **OR** “match schedule*” **OR** competit* **OR “**competitive performance” **OR** “competitive demand*” **OR** “competitive matches” **OR** “competitive season” **OR** “competition schedule” **OR** game* **OR** “game play*” **OR** “game activit*” **OR** “game analysis” **OR** “game performance*” **OR** “game demand*” **OR** “game intensit*”
Search phase	1 **AND** 2 **AND** 3

### Screening Strategy and Study Selection

All electronic search results were initially exported to Microsoft Excel (Microsoft, Redmond, WA, USA) by the lead author (DH). Identification of eligible studies followed a three-stage process. First, duplicate studies were removed (DH). Second, studies that were clearly ‘out of scope’ were excluded following screening of the title and abstract (DH)—if a clear decision could not be made at this stage, studies were taken forward. The final stage was completed independently by two authors (DH, CC) and involved removal of studies by the exclusion criteria following screening of the full text. Any discrepancies (*n* = 13) on the final inclusion-exclusion status were resolved by consensus discussion.

### Data Extraction

All data were extracted into a custom-made Microsoft Excel sheet by one author (DH). During the data extraction process, studies that used the same data across multiple studies were excluded, with only the earliest publication date used. This resulted in the exclusion of a further five [14, 29–32] studies (Table 2, exclusion criteria: #10). Data extracted were organised according to the sample studied (sport, position, age, body mass, stature), competition details (type, year, number of matches, data files) and classification of ‘eliteness’ (semi-elite, competitive elite, successful elite, world-class elite). The classification of ‘eliteness’ given to each study sample was undertaken independently by two authors (DH, CC) using a modified version of the model (Table S1 of the Electronic Supplementary Material [ESM]) and classification (Table S2 of the ESM) proposed by Swann et al. [33], which allows consistent within- and between-sport comparisons to be made. Any discrepancies were resolved by consensus discussion before the final classification was given (Table S3 of the ESM).

**Table 2:** Study inclusion–exclusion criteria

	Inclusion criteria	Exclusion criteria
1	Original research articles	Reviews, magazines, surveys, opinion pieces, books, periodicals, editorials, conference abstracts, non-academic/non-peer-reviewed text
2	Field-based team sports or court-based invasion games	Striking and fielding games (cricket, baseball), net and wall games (badminton, tennis, volleyball) and ice-, sand- or water-based team sports
3	Competitive able-bodied elite athletes^a^	Athletes with physical or mental disability, athletes competing outside of the top 3 tiers in their sport, match officials
4	Participants with mean age ≥ 18 years	Participants with mean age < 18 years
5	Competitive match play rules (i.e. full-sized court/pitch, regulation number of players)	Training and small-sided games, non-competitive matches (friendlies), match simulations
6	GPS systems (with sampling frequency ≥ 5 Hz)	GPS units (with sampling frequency of < 5 Hz), any non-GPS system (e.g. digital video-based tracking)
7	Reported both higher (> 2.5 m·s^−2^) intensity acceleration and deceleration events separately and concurrently	Reported just acceleration or deceleration events in isolation, combined acceleration and deceleration variables into one metric (e.g. average acceleration, velocity change load, acceleration load, high-intensity efforts, explosive distance), no high-intensity thresholds reported, did not report acceleration or deceleration events (i.e. focus was on other locomotor related variables, e.g. sprinting, high-intensity running, metabolic power)
8	Reported data for full match duration^b^	Reported only part of a match (i.e. first half, extra-time)
9	Full text available in English	Cannot access full text in English
10	Data set used in one study^c^	Studies using the same data set from an earlier publication (salami slicing)

*GPS* global positioning system

^a^Elite athletes classified using a modified version of Swann et al. [33] (see Table S3 of the Electronic Supplementary Material)

^b^Match duration greater than 75%

^c^Study with earliest publication date used when multiple studies published using same data set

In line with recent recommendations [34, 35] for the collecting, processing and reporting of data from GPS devices, we also recorded the device brand and model details, software version, sampling frequency (Hz), minimal effort duration (MED), number of satellites used and horizontal dilution of precision. These guidelines were also used to produce a checklist (Table S4 of the ESM) that helped to inform judgements (Table S5 of the ESM) on the risk of bias (RoB) for each included study within the areas of ‘data collection’, ‘data processing’ and ‘normative profile’ (further information in Sect. 2.6).

The mean, standard deviation and number of observations (match data files) were extracted for all acceleration and deceleration events and also categorised according to the temporal profile (first half, second half, full match), measurement approach (absolute or relative: number of efforts, distance covered, time spent) and intensity threshold (m·s^−2^) used to delineate the occurrence of a high and very high intensity acceleration and deceleration.

### Missing Data

If the mean, standard deviation (SD) and number of data files could not be obtained from published records, the corresponding authors [17, 18, 20, 36] were contacted (via e-mail, social media) for further information. If the corresponding authors could not provide data for the full match, but periods of play had been reported (first and second half), then the full match mean and SD were calculated using the formula for combining group data as recommended in the Cochrane guidelines [27]:

$${\text{Combined}}\;{\text{group}}\;{\text{mean}} = \frac{{(N_{1} M_{1} ) + (N_{2} M_{2} )}}{{(N_{1} + N_{2} )}},$$

where *N* equals the number of data files and *M* equals the mean number of accelerations or decelerations for each group.

$${\text{Combined}}\;{\text{group}}\;{\text{SD}} = \sqrt {\frac{{\left( {{\text{SD}}_{1}^{2} + {\text{SD}}_{2}^{2} } \right)}}{2}} ,$$

where SD equals the standard deviation for the number of accelerations and decelerations completed for each group.

Combined means and SDs were only used for one study [18] that reported relative acceleration and deceleration events (i.e. per minute).

### Assessment of Risk of Bias

In accordance with Cochrane collaboration guidelines, a ‘domain-based’ evaluation was undertaken, in which critical assessments are made to inform a judgement about the overall RoB for each included study [27]. Numerous methodological factors associated with GPS devices have been shown to influence the quantification of acceleration and deceleration events during match play [34, 35]. Furthermore, a range of contextual, tactical and fatigue-related factors, amongst others, may influence match running profiles in team sports [37]. Therefore, the domain most relevant to the outcomes of this review was ‘detection bias’, which appraises the systematic differences between groups in how outcomes are determined [27]. First, using recent guidelines [34, 35] a checklist was produced (Table S4 of the ESM) that identified key entries (‘data collection’, ‘data processing’, ‘normative profile’) and associated criteria that could be used to facilitate overall judgement (Table S5 of the ESM) about RoB for each individual entry. Two reviewers (DH, CC) independently completed the checklist using six response options: (1) ‘yes’, (2) ‘no’, (3) ‘no information’, (4) ‘not applicable’, (5) ‘probably yes’ and (6) ‘probably no’ as recommended by the Cochrane Collaboration guidelines [27]. A final judgement (Table S4 of the ESM) about RoB for each key entry was then made by each reviewer (DH, CC) using three possible outcomes: (1) low RoB: plausible bias unlikely to seriously alter the results; (2) unclear RoB: plausible bias that raises some doubts about the results; and (3) high RoB: plausible bias that seriously weakens confidence in the results [27]. The inter-rater agreement (kappa) was 0.63 (quality control), 0.79 (event identification) and 1.00 (normative profile), which are considered to be good to excellent magnitudes of agreement [27]. Any discrepancies in the final judgement of RoB between reviewers were resolved by consensus discussion.

### Data Analysis and Interpretation of Results

A meta-analysis was performed using Review Manager Software for Mac (RevMan 5.2; Cochrane Collaboration, Oxford, UK). The inverse random-effects model for continuous data was used for statistical analysis because it accounts for heterogeneity of the included studies [27]. The meta-analysis sought to compare full match sport and positional differences in the frequency of high-intensity accelerations and decelerations. The type of sport was considered a priori to be a key moderating variable because significant differences in match-activity profiles between field-based sports have been shown to exist, even when accounting for differences in match duration [38]. To illustrate temporal changes in acceleration and deceleration outputs from the first to the second half periods of match play, a further two meta-analyses were completed, with the different intensity thresholds (‘high’ and ‘very high’) used as sub-groups.

One author (DH) entered the mean, SD and total number of observations for each separate meta-analysis. The effect magnitude was calculated using the standardised mean difference (SMD) alongside 95% confidence intervals (CIs) and presented in forest plots using GraphPad software (Prism 7, GraphPad Software Inc., La Jolla, CA, USA). The SMD includes a correction (Hedges’s *g*) for small sample bias and expresses results in a uniform scale despite differences in how the outcome variable was measured [27]. The SMD was interpreted with a qualitative scale using the thresholds outlined by Hopkins et al. [39]: < 0.2 = trivial; 0.2–0.6 = small; 0.6–1.2 = moderate; 1.2–2.0 = large; 2.0–4.0 = very large; and > 4.0 = extremely large. The percentage of total variation between and within subgroups due to heterogeneity was measured using the *I*^2^ statistic for quantifying inconsistency in study results [40]. The magnitude of inconsistency was interpreted according to the criteria of Higgins et al. [40]: low (0–25%), moderate (26–74%) and high (75–100%). *P* < 0.05 was considered statistically significant.

## Results

### Search Results

**Fig. 1:**
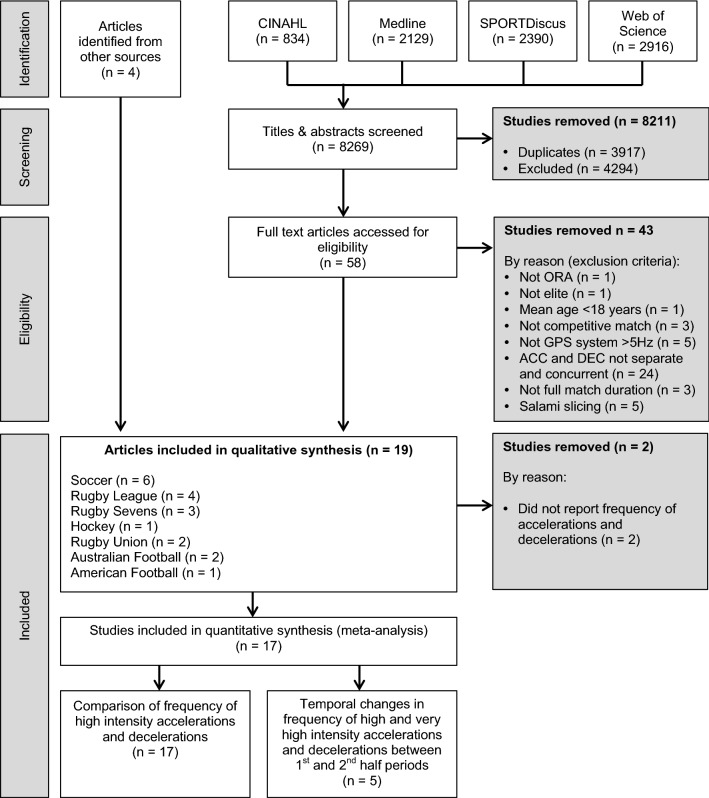
Step-by-step process leading to the identification of studies eligible for a systematic review. *ACC* Acceleration, *DEC* Deceleration, *GPS* global positioning system, *ORA* original research article

The initial search identified 8269 articles across four databases (CINAHL = 834, MEDLINE = 2129, SPORTDiscus = 2390, Web of Science = 2916). Then 8211 studies were removed following screening of the study title and abstract because of duplication (*n* = 3917) or not meeting the inclusion criteria (*n* = 4294). A further 43 records were removed using the exclusion criteria after screening the full text, resulting in 15 studies that met the inclusion criteria. A further four studies that met the inclusion criteria were identified from other sources, resulting in 19 studies meeting the inclusion criteria. Two of these studies [16, 41] were not considered for the meta-analysis because of reporting distance and time-related variables only, but was included in the descriptive qualitative synthesis. This resulted in 17 independent studies that provided 115 estimates being included in the meta-analysis (Fig. 1). From these 17 studies, 99 estimates were used to examine the differences in the frequency of high and very high intensity accelerations vs. decelerations in competitive match play. The remaining 16 estimates obtained from five of these studies were used to examine the temporal changes in high and very high intensity accelerations and decelerations from the first to the second half periods of match play.

### Study Characteristics

The characteristics of the 19 included studies are summarised in Table 3. One study investigated American Football [42], two Australian Football [43, 44], one hockey [18], four rugby league [29, 45–47], three rugby sevens [36, 48, 49], two rugby union [41, 50] and six soccer [10, 16, 17, 19, 20, 51]. Across all seven team sports investigated, the total sample included 469 players with a mean age ranging from 18 to 29 years. No studies investigated high-intensity accelerations and decelerations in female players. The samples of male players across all sports were classified as competitive elite (*n* = 1, 5%), successful elite (*n* = 8, 40%) and world-class elite (*n* = 11, 55%). One study [50] reported data from two different samples of elitism.

**Table 3:** Characteristics of the included studies

Study	Sport	Position (*n*)	Sample				Competition details				Classification of eliteness
			*n*	Age (years)	Body mass (kg)	Stature (cm)	Type	Year	Matches (*n*)	Files (*n*)	
Coutts et al. [43]	AuF	TB	39	25 ± 3	89 ± 9	188 ± 7	Australian Football League	NR, 2 seasons	19	35	Successful elite
		MB								70	
		MID								145	
		TF								23	
		MF								48	
		RKS								21	
Johnston et al. [44]	AuF	MID	30	24 ± 3	89 ± 9	187 ± 7	Australian Football League	2011–2012	1–29	278	Successful elite
		FF								31	
		FD								86	
		RKS								24	
Wellman et al. [42]	AmF	WR	33	21 ± 1	91 ± 12	186 ± 11	NCAA Division 1	2014	12	41	World class elite
		RB			98 ± 10	182 ± 2				41	
		QB			93 ± 2	192 ± 2				12	
		TE			115 ± 7	197 ± 1				21	
		OL			137 ± 5	192 ± 4				38	
		DB			86 ± 6	183 ± 5				55	
		DT			135 ± 0	191 ± 0				17	
		DE			119 ± 6	193 ± 4				33	
		LB			106 ± 3	186 ± 3				36	
Morencos et al. [18]	HK	BK (5)	16	26 ± 3	75 ± 6	177 ± 5	Spanish Hockey Premier League	NR, 2 seasons	17	45	Competitive elite
		MID (6)								42	
		FOR (5)								26	
Cummins et al. [29]	RL	ADJ (4)	18	25 ± 4	99 ± 7	185 ± 7	National Rugby League	2013	NR	74	World class elite
		HUF (3)								36	
		OB (4)								59	
		WRF (7)								104	
Dempsey et al. [45]	RL	FOR (37)	57	30 ± 4	103 ± 7	188 ± 5	Four Nations	2011–12	6	37	World class elite
		BK (20)		26 ± 4	92 ± 6	182 ± 6				20	
Kempton et al. [46]	RL	ADJ	25	25 ± 4	99 ± 8	185 ± 6	National Rugby League	2010–11	39	118	World class elite
		HUF								52	
		OB								121	
		WRF								93	
Oxendale et al. [47]	RL	BK	17	25 ± 4	99 ± 10	184 ± 6	English Super League	2014	4	11	World class elite
		FOR								17	
Furlan et al. [49]	RS	Team	12	22 ± 3	90 ± 9	185 ± 6	IRB World Series	2013–14	6	21	Successful elite
Higham et al. [48]	RS	Team	19	21 ± 3	90 ± 7	181 ± 5	IRB World Series	NR	11	75	Successful elite
							Domestic		16	99	
Suarez-Arrones et al. [36]	RS	Team	12	27 ± 2	86 ± 9	182 ± 7	2 International tournaments	NR	NR	30	Successful elite
Cunningham et al. [50], senior	RU	FR	27	26 ± 2	119 ± 5	186 ± 4	Six Nations	2014–15	8	97	World class elite
		SR		26 ± 3	117 ± 5	199 ± 2					
		BR		26 ± 3	118 ± 10	190 ± 3					
		HB		24 ±3	89 ± 5	180 ± 6					
		CTR		26 ± 1	102 ± 7	190 ± 4					
		B3		25 ± 3	92 ± 2	184 ± 4					
Cunningham et al. [50], under 20	RU	FR	43	20 ± 1	112 ± 6	184 ± 3	Six Nations; Junior World Cup	2014–15	15	161	Successful elite
		SR		20 ± 1	115 ± 4	200 ± 2					
		BR		20 ± 0	102 ± 4	188 ± 3					
		HB		20 ± 0	84 ± 4	176 ± 2					
		CTR		20 ± 1	96 ± 7	183 ± 5					
		B3		20 ± 1	90 ± 5	184 ± 4					
Jones et al. [41]	RU	Team	33	25 ± 4	104 ± 11	NR	European Cup; Celtic League	2012–13	13	71	World class elite
Akenhead et al. [16]	SOC	Team	36	19 ± 1	80 ± 7	183 ± 5	English Premier League Reserve	2010–11	18	648	World class elite
De Hoyo et al. [10]	SOC	Team	7	18 ± 1	76 ± 7	180 ± 2	Spanish First League	NR	1	7	World class elite
Russell et al. [19]	SOC	Team	5	21 ± 1	70 ± 2	177 ± 3	English PL Reserve Team	2013	1	5	World class elite
Russell et al. [17]	SOC	Team	11	20 ± 1	71 ± 4	180 ± 10	English Premier League Reserve	2013–14	19 (6 ± 4 per player)	76	World class elite
Tierney et al. [51]	SOC	WD (10)	46	20 ± 3	80 ± 6	179 ± 5	Under 21 and Under 18 English Football League	2014–15	42	420	Successful elite
		CD (9)								378	
		WM (9)								378	
		CM (10)								420	
		FW (8)								336	
Wehbe et al. [20]	SOC	DEF (6)	19	26 ± 5	80 ± 5	183 ± 5	Australian A-League	2011–12	8	48	Successful elite
		MID (9)		26 ± 6	75 ± 4	178 ± 5				54	
		FOR (4)		26 ± 5	81 ± 4	183 ± 7				32	

Data are presented as mean ± standard deviation

*ADJ* adjustable, *AmF* American football, *AuF* Australian football, *B3* back three, *BK* back, *BR* back row, *CD* central defender, *CM* central midfielder, *CTR* centre, *DB* defensive back, *DE* defensive end, *DEF* defender, *DT* defensive tackle, *FD* fixed defender, *FF* fixed forward, *FOR* forward, *FR* front row, *HB* half back, *HK* hockey, *HUF* hit-up forward, *IRB* international rugby board, *LB* linebacker, *MB* mobile backs, *MID* midfielders, *MF* mobile forwards, *NCAA* National Collegiate Athletic Association, *NR* not reported, *OB* outside back, *OL* offensive linesman, *QB* quarter back, *RB* running back, *RKS* rucks, *RL* rugby league, *RS* rugby sevens, *RU* rugby union, *SOC* soccer, *SR* second row, *TB* tall backs, *TF* tall forwards, *TE* tight end, *WD* wide defender, *WM* wide midfielder, *WR* wide receiver, *WRF* wide-running forward

### Measurement of High-Intensity Accelerations and Decelerations

Table 4 illustrates the different methodologies used across studies to measure high-intensity accelerations and decelerations during match play. Almost half of the included studies in this review used the brand GPSports (47%, *n* = 9), while 32% (*n* = 6) used Catapult Sports and 21% (*n *= 4) used STATSports. Sixty-three percent (*n* = 12) of studies used GPS with a raw 10-Hz sampling frequency, with the remaining 32% (*n *= 7) of studies using 5 Hz. Four of the studies [29, 36, 42, 49] that captured data at 5 Hz incorporated an interpolation algorithm that resulted in a 15-Hz output. The MED used to delineate the minimal time required to be above the specified high-intensity acceleration or deceleration threshold for an effort to be recorded was reported in four studies [17, 36, 43, 46] and ranged between 0.2 and 1 s. The number of satellites used to infer GPS signal quality was reported in four studies [16, 44, 46, 50] and ranged from 4 to 13. Horizontal dilution of precision used to indicate the accuracy of the GPS horizontal positional signal was reported in two studies [16, 44] and values ranged from 0.8 to 1. The most common threshold used to classify the start of high-intensity acceleration or deceleration was 3 m·s^−2^ (*n* = 11, 58%). Six studies [20, 36, 42, 48–50] also used a very high intensity threshold starting at either 3.5 m·s^−2^ (*n* = 1) or 4 m·s^−2^ (*n* = 5). Variables used to report high or very high intensity acceleration and decelerations included frequency (*n* = 17 studies), distance covered (*n* = 3 studies) and time spent (*n* = 1 study). Sixteen studies reported data in absolute terms (total match duration), whilst five studies reported these variables relative to time (i.e. number per minute). Only one study [45] reported data using both absolute and relative formats.

**Table 4:** Summary of the methodological procedures used to measure high and very high intensity accelerations and decelerations using global positioning system (GPS) with overall risk of bias judgements

Study	GPS device		Data collection					Data processing				Thresholds (m·s^−2^)		Risk of bias		
	Brand	Model	Software version	SF (Hz)	SAT (*n*)	HDP (*n*)	Variables measured	MED (s)			Raw/software	High	Very high	*A*	*B*	*C*
Australian Football																
Coutts et al. [43]	Catapult Sports	NI	Sprint v5.0.6	10	NR	NR	*F* (n)		0.2		Raw	> 2.78		?	+	+
Johnston et al. [44]	Catapult Sports	MinimaxX S3 and S4	Sprint v5.0.9	5 and 10	12	1	*F* (n)		NR		Software	> 2.78		+	?	+
							*D* (m)									
							*T* (s)									
American Football																
Wellman et al. [42]	GPSports	SPI HPU	Team AMS	5^a^	NR	NR	*F* (n)		NI NR		Software	2.6–3.5	> 3.5	?	?	+
Hockey																
Morencos et al. [18]	GPSports	SPI Elite	Team AMS v.R1.215.3	10	NR	NR	*F* (n·min^−1^)		NR		Software	> 3		−	?	+
Rugby league																
Cummins et al. [29]	GPSports	SPI-ProX	NI	5^a^	NR	NR	*F* (n·min^−1^)			NR	Raw	> 2.78		−	?	?
Dempsey et al. [45]	GPSports	SPI-ProX	Team AMS vR1 2012.4	10	NR	NR	*F* (n)			NR	Software	> 3		?	?	?
							*F* (n·min^−1^)									
Kempton et al. [46]	GPSports	SPI-Pro	Team AMS vR1 2012.1	5	9	NR	*F* (n)			0.4	Raw	> 2.78		?	+	+
Oxendale et al. [47]	Catapult Sports	MinimaxX	Team 2.5	10	NR	NR	*F* (n)			NR	NR	> 2.79		−	?	?
Rugby sevens																
Furlan et al. [49]	GPSports	SPI-HPU	Labview 2011^b^	5^a^	NR	NR	*F* (n·min^−1^)			NR	Raw	3–4	> 4	−	?	−
Higham et al. [48]	Catapult Sports	MinimaxX	Team Sport v2.5	5	NR	NR	*F* (n·min^−1^)			NR	Software		> 4	−	+	?
Suarez-Arrones et al. [36]	GPSports	SPI-ProX	Team AMS R1 2013.9	5^a^	NR	NR	*F* (n)			1	Software	> 2.78	> 4	−	+	−
Rugby union																
Cunningham et al. [50] U20	STATSports	Viper Pod	Viper PSA	10	4	NR	*F* (n)			NR	Software	3–4	> 4	+	?	+
Cunningham et al. [50] Senior	STATSports	Viper Pod	Viper PSA	10	4	NR	*F* (n)			NR	Software	3–4	> 4	+	?	?
Jones et al. [41]	Catapult Sports	MinimaxX v4.0	Sprint	10	NR	NR	*D* (m)			NR	Software	> 3		?	?	?
Soccer																
Akenhead et al. [16]	Catapult Sports	MinimaxX	Logan Plus v4.5	10	13	0.8	*D* (m)			NR	Raw	> 3		+	?	?
De Hoyo et al. [10]	GPSports	SPI Elite	Team AMS	10	NR	NR	*F* (n)			NR	Software	> 3		?	?	−
Russell et al. [19]	STATSports	Viper Pod	Viper PSA	10	NR	NR	*F* (n)			0.5	Software	> 3		?	+	−
Russell et al. [17]	STATSports	Viper Pod	Viper PSA	10	NR	NR	*F* (n)			0.5	Software	> 3		?	+	−
Tierney et al. [51]	STATSports	NI	NI	10	NR	NR	*F* (n)			NR	NR	> 3		−	−	+
Wehbe et al. [20]	GPSports	SPI-Pro	NI	5	NR	NR	*F* (n)			0.5	NR	2.5–4	> 4	−	?	?

*A* data collection; *B* data processing, *C* normative profile, *D* distance, *F* frequency, *HDP* horizontal dilution of precision, *MED* minimal effort duration, *NR* not reported, *SAT* number of satellites, *SF* sampling frequency, *T* time, *+* indicates low risk of bias (plausible bias unlikely to seriously alter the results), ? indicates unclear risk of bias (plausible bias that raises some doubt about the results), − indicates high risk of bias (plausible bias that seriously weakens confidence in the results)

^a^Interpolated to 15 Hz from 5-Hz GPS raw velocity data

^b^Custom written software

### Risk of Bias

**Fig. 2:**
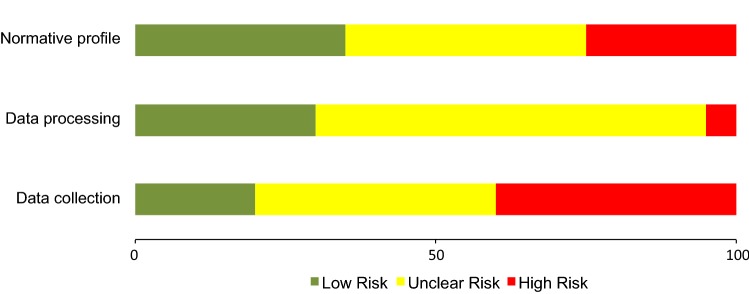
Risk of bias graph

The overall RoB judgement (low, unclear and high) for each key entry (data collection, data processing and normative profile) and for each individual study is reported in Table 4. Across all studies, the greatest RoB (40% high RoB) was observed in the data collection domain (Fig. 2), as the majority of studies did not report the number of satellites obtained (85%) or the horizontal dilution of precision (90%). Notably, within this entry, 70% (*n* = 14) of the studies used a GPS device with a 10-Hz sampling frequency. The greatest amount of uncertainty (65%) was in the data processing domain, as only eight studies [17, 19, 20, 36, 43, 46–48] reported the MED. The lowest risk of bias (35% low RoB) was the normative profile domain, in which nearly half (45%, *n *= 9) of the studies [16, 18, 41–44, 46, 48, 50, 51] used greater than ten matches in total, and over half (60%, *n* = 12) of the studies [18, 20, 29, 42–47, 50, 51] reported position-specific acceleration and deceleration data. The number of matches used to characterise the average high-intensity acceleration and deceleration demands ranged between 1 and 42.

### Meta-Analysis: Frequency of High-Intensity Accelerations and Decelerations

**Fig. 3:**
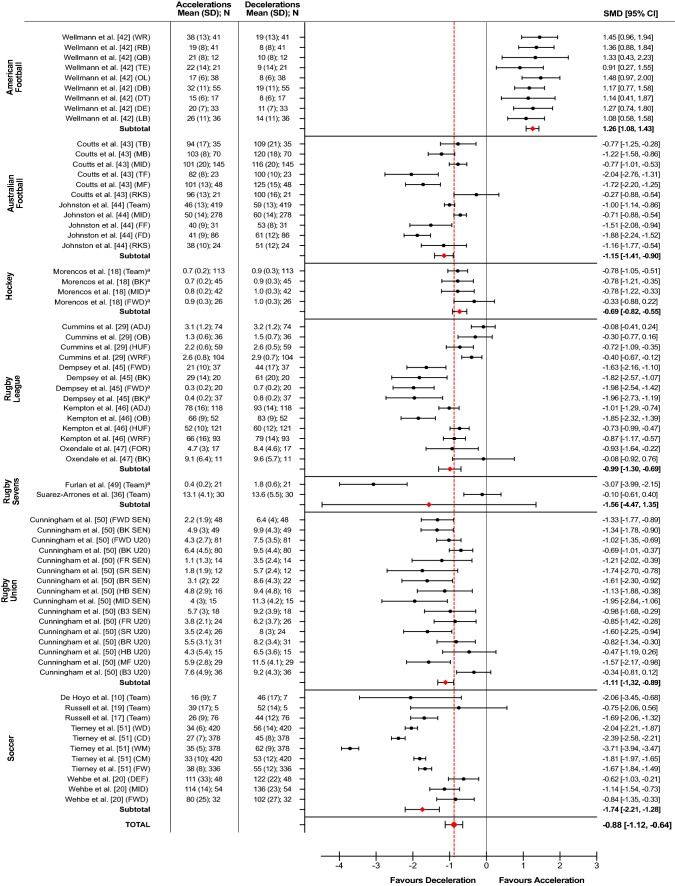
Forest plot displaying the standardized mean differences (SMD) and 95% confidence intervals (CIs) in the frequency of high (> 2.5 m**·**s^−2^) intensity accelerations vs. decelerations in elite team sports competitive match play. ^a^Frequency relative to time (n**·**min^−1^). *ADJ* adjustable, *B3* back three, *BK* back, *BR* back row, *CD* central defender, *CM* central midfielder, *CTR* centre, *DB* defensive back, *DE* defensive end, *DEF* defender, *DT* defensive tackle, *FD* fixed defender, *FF* fixed forward, *FR* front row, *FWD* forward, *HB* half back, *HUF* hit-up forward, *MB* mobile backs, *MF* mobile forwards, *MID* midfielder/centre, *LB* linebacker, *OB* outside back, *OL* offensive linesman, *QB* quarter back, *RB* running back*, RKS* rucks, *SD* standard deviation, *SEN* senior, *SR* second row, *TB* tall backs, *TE* tight end, *TF* tall forwards, *U20* under 20, *WD* wide defender, *WM* wide midfielder, *WR* wide receiver, *WRF* wide-running forward

Sixteen studies (5220 files, 67 SMD) across seven sports: American Football (294 files, 9 SMD), Australian Football (1180 files, 11 SMD), hockey (226 files, 4 SMD), rugby league (799 files, 14 SMD), rugby sevens (51 files, 2 SMD), rugby union (516 files, 16 SMD) and soccer (2154, 11 SMD) reported the frequency of high-intensity accelerations and deceleration events (Fig. 3). An heterogeneity analysis showed a significant high percentage of total variation (*p* < 0.00001, *I*^2^ = 99%) between sports (Table 5). Only American Football demonstrated a higher frequency of high-intensity accelerations compared to decelerations (SMD = 1.26, 95% CI 1.06–1.43). All other sports had a greater frequency of high-intensity decelerations compared to accelerations with SMD ranging from moderate (− 0.69) in hockey to large (− 1.74) in soccer. The percentage of total variation amongst estimates in American Football and hockey was low (*p* = 0.51–0.9, *I*^2^ = 0%). In all other sports, a significant moderate to high percentage of total variation was evident (*p* < 0.003, *I*^2^ = 57–97%).

**Table 5:** Effect of heterogeneity across included studies within each meta-analysis

Meta-analysis	Sub-group	Number of estimates	Number of GPS files	Between-group *I*^2^ (%)	Within-group *I*^2^ (%)	Qualitative descriptor	*P* value
Frequency of high-intensity accelerations and decelerations		67	5220	99		High	< 0.00001
	American Football	9	294		0	Low	0.9
	Australian Football	11	1180		85	High	< 0.0001
	Hockey	4	226		0	Low	0.51
	Rugby league	14	799		87	High	< 0.0001
	Rugby sevens	2	51		97	High	< 0.0001
	Rugby union	16	516		57	Moderate	0.003
	Soccer	11	2154		97	High	< 0.0001
Frequency of very high intensity accelerations and decelerations		32	1169	94		High	< 0.00001
	American Football	9	294		92	High	< 0.00001
	Rugby sevens	4	225		95	High	< 0.00001
	Rugby union	16	516		31	Moderate	0.11
	Soccer	3	134		89	High	< 0.00001
Temporal changes in frequency of accelerations		8	373	0		Low	0.93
	High intensity	5	227		0	Low	0.45
	Very high intensity	3	146		0	Low	0.82
Temporal changes in frequency of decelerations		8	373	0		Low	0.72
	High intensity	5	227		53	Moderate	0.08
	Very high intensity	3	146		34	Moderate	0.22

*GPS* global positioning system

### Meta-Analysis: Frequency of Very High Intensity Accelerations and Decelerations

**Fig. 4:**
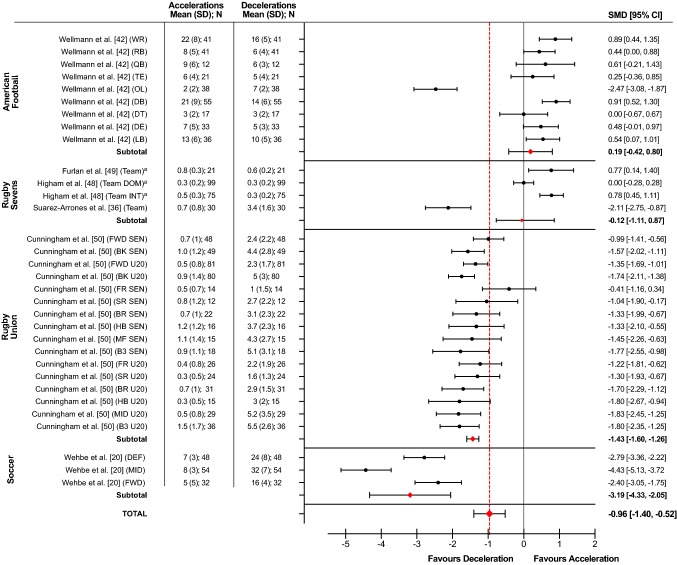
Forest plot displaying the standardized mean differences (SMD) and 95% confidence intervals (CIs) in the frequency of very high (> 3.5 m·s^−2^) intensity accelerations vs.decelerations in elite team sports competitive match play. ^a^Frequency relative to time (n·min^−1^). *B3* back three, *BK* back, *BR* back row, *DB* defensive back, *DE* defensive end, *DEF* defender, *DOM* domestic, *DT* defensive tackle, *FR* front row, *FWD* forward, *HB* half back, *INT* international, *LB* linebacker, *MID* midfielder/centres, *QB* quarter back, *OL* offensive linesman, *RB* running back*, SD* standard deviation, *SEN* senior, *SR* second row, *TE* tight end*, U20* under 20s, *WR* wide receiver

Six studies (1169 files, 32 SMD) across four sports: American Football (294 files, 9 SMD), rugby sevens (225 files, 4 SMD), rugby union (516 files, 16 SMD) and soccer (134, 3 SMD) reported the frequency of very high intensity accelerations and decelerations (Fig. 4). An heterogeneity analysis showed a significantly high percentage of total variation (*p* < 0.00001, *I*^2^ = 94%) between sports (Table 5). Only American Football had a greater frequency of very high intensity accelerations compared to decelerations (SMD = 0.19, 95% CI − 0.42 to 0.80), although the difference was trivial. All other sports had a greater frequency of very high intensity decelerations compared to accelerations with SMD ranging from trivial (− 0.12) in rugby sevens to very large (− 3.19) in soccer. The percentage of total variation across studies and positional roles ranged from moderate (*p* = 0.11, *I*^2^ = 31%) in rugby union to high (*p* < 0.00001, *I*^2^ = 89–95%) in all other sports.

### Meta-Analysis: Temporal Changes

#### High and Very High Intensity Accelerations: Temporal Changes

**Fig. 5:**
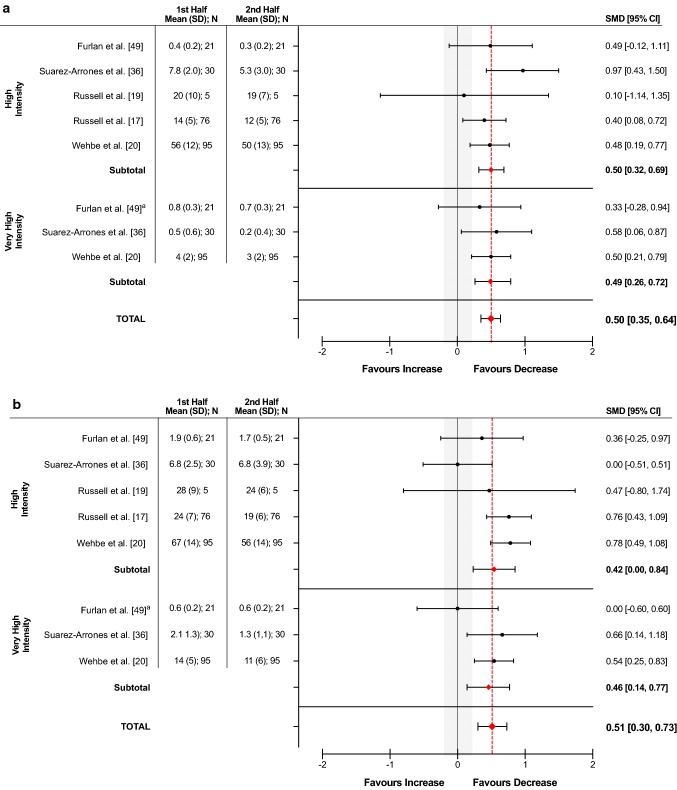
Forest plots displaying the standardized mean difference (SMD) and 95% confidence intervals (CIs) in the **a** temporal changes in the frequency of high (> 2.5 m**·**s^−2^) and very high (> 3.5 m**·**s^−2^) intensity accelerations and **b** high (> 2.5 m**·**s^−2^) and very high (> 3.5 m**·**s^−2^) intensity decelerations from the first to the second half periods of match play. ^a^Frequency relative to time (n**·**min^−1^). *SD* standard deviation

Five studies [17, 19, 20, 36, 49] covering two sports (rugby sevens, soccer) reported temporal changes in the frequency of high and very high intensity accelerations between the first and second half periods of match play. There was a low percentage (*p* = 0.45–0.93, *I*^2^ = 0%) of total variation due to heterogeneity between and within the high and very high intensity subgroups (Table 5). The SMD for both high (0.50) and very high intensity (0.49) sub-groups showed a small decrease in the frequency of accelerations completed from the first to the second half periods of match play (Fig. 5a). In rugby sevens, the SMD ranged between small (0.33) to moderate (0.97), whilst in soccer the decrease was small (SMD = 0.10–0.50).

#### High and Very High Intensity Decelerations: Temporal Changes

Five studies [17, 19, 20, 36, 49] covering two sports (rugby sevens, soccer) reported temporal changes in high and very high intensity decelerations between the first and second half periods of match play. There was a low percentage (*p* = 0.72, *I*^2^ = 0%) of total variation due to heterogeneity between the high and very high intensity subgroups. Within the high and very high-intensity subgroups, a moderate percentage (*p* = 0.08–0.22, *I*^2^ = 34–53%) of total variation due to heterogeneity was evident across studies (Table 5). The SMD for both high (0.54) and very high intensity (0.46) sub-groups showed a small decrease in the frequency of decelerations performed from the first to the second half periods of match play (Fig. 5b). In rugby sevens, the SMD ranged between trivial (0.00) and moderate (0.66), whilst in soccer it ranged between small (SMD = 0.47) and moderate (0.78).

### Descriptive Analysis: Distances and Time Spent Accelerating and Decelerating

**Fig. 6:**
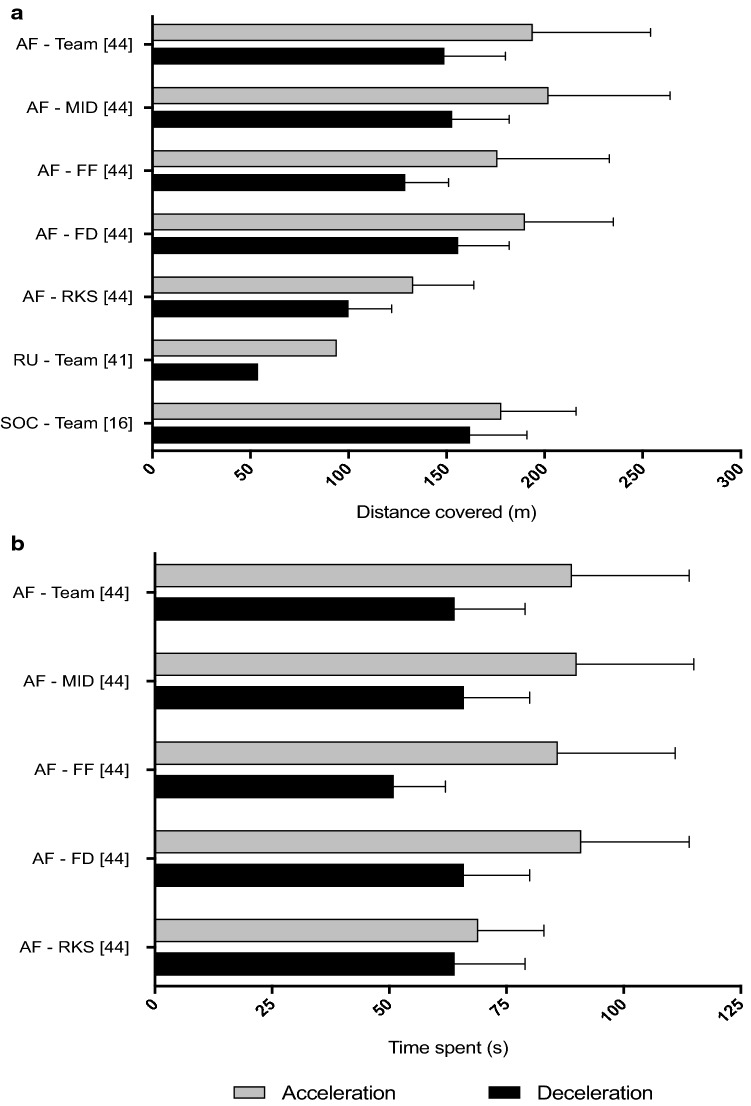
**a** Distances and **b** times spent accelerating and decelerating at high intensity during elite competitive match play. *AF* Australian football, *FF* fixed forward, *FD* fixed defender, *MID* midfielder, *RKS* ruckman, *RU* rugby union, *SOC* soccer

Three studies investigating Australian Football [44], rugby union [41] and soccer [16] reported the absolute distance spent accelerating and decelerating at high intensity (Fig. 6a). Only the study by Johnston et al. [44] reported positional differences. Australian Football had the highest full match distance (194 m) spent accelerating at high intensity followed by soccer (178 m) and rugby union (94 m). In Australian Football, midfielders reported the highest total match distance (202 m) spent accelerating at high intensity, followed by fixed defenders (190 m), fixed forwards (176 m) and rucks (133 m). Soccer had the highest full match distance (162 m) spent decelerating at high intensity, followed by Australian Football (149 m) and rugby union (54 m).

Only one study [44] investigating Australian Football reported the time spent accelerating and decelerating at high intensity (Fig. 6b). On average, all players spent a longer time (89 s) accelerating at high intensity compared to decelerating (64 s). Fixed defenders and midfielders spent the longest time accelerating (91 and 90 s, respectively) and decelerating (66 and 66 s, respectively) at high intensity amongst all positional roles. Rucks had the lowest time (69 s) spent accelerating, whilst fixed forwards had the lowest time (51 s) spent decelerating. No studies reported either the distance or time spent accelerating or decelerating at very high intensities.

## Discussion

To our knowledge, this is the first meta-analysis to compare differences between the most intense (> 2.5 m·s^−2^) accelerations and decelerations in elite team sports competitive match play. Over half (55%) of the included studies investigated players classified as ‘world-class’ elite. As such, this review provides high-performance practitioners with novel insights into the acceleration and deceleration demands of players at the highest standards of match play. Using recent guidelines [34, 35], a secondary aim was to review current methodological limitations around the measurement of high-intensity accelerations and decelerations, with a view to recommending future directions. The main finding from our meta-analysis is that American Football was the only sport with a higher frequency of high (SMD = 1.26) and very high (SMD = 0.19) intensity accelerations compared to decelerations. In all other sports, there was a greater frequency of high (SMD = − 0.48 to − 1.74) and very high (SMD = − 0.32 to − 3.19) intensity decelerations compared to accelerations.

### Frequency of High-Intensity Accelerations Compared to Decelerations

In all team sports apart from American Football, there was a greater frequency of high-intensity decelerations compared to accelerations (SMD = − 0.69 to − 1.74). The largest difference (SMD = − 1.74) was found in soccer, although a significant high variation (*I*^2^ = 97%) was evident between teams and positional estimates. The evolution of elite soccer match play requires contemporary players to perform more short high-intensity actions to fulfil tactical responsibilities, whilst in and out of possession, and during ball possession transitions [3, 52]. Whilst these studies have shown an evolutionary progression in the frequency of rapid accelerations, the findings of our meta-analysis illustrate the prevalence of high-intensity decelerations to soccer match-play performance. Although high-intensity decelerations have been shown to be very short in duration (72% less than 1 s duration) [53], they comprise the highest magnitude of mechanical load per metre—reportedly up to 65% greater than any other match-play activity and around 37% more than similarly intense accelerations [7]. Even in elite players, this load places a significant demand on the ability to repeatedly absorb high eccentric braking forces, of which the cumulative effect following match play is associated with markers of exercise-induced muscle damage [12, 54], deficits in countermovement jump concentric and eccentric phase performance [10, 12], and asymmetry in hamstring isometric strength [55], effects that have been shown to last up to 64 hours post-match. The muscle damage resulting from repeated intense decelerations is caused by strain to muscle fibres during eccentric (lengthening) contractions that result in disruption of the integrity of muscle cells [56]. Unlike maximum voluntary force that can also be affected by concentric exercise (metabolic fatigue), the rate of force development is particularly affected by muscle damage resulting in a diminished capacity to both produce and attenuate forces in very short time periods that is commonly required to enhance sports performance and reduce injury risk [57, 58]. Specific attention to loading strategies that can “mechanically protect” players from these damaging consequences of high-intensity decelerations are necessary [9]. For example, studies examining the repeated bout effect have shown that greater resistance to muscle damage can be obtained by prior eccentric or isometric exercise [59, 60]. Implementing such strategies in preparation for match play could attenuate the amount of damage accumulated per deceleration effort, resulting in less mechanical fatigue and a reduced risk of tissue failure occurring (this representing an increased ‘deceleration efficiency’) [61].

Our findings also highlight significant team and positional differences in the frequency of high-intensity decelerations compared to accelerations in soccer. In a study by Tierney et al. [51], wide midfielders completed the most high-intensity decelerations (*n* = 62) of any position, and also had the highest difference (SMD = − 3.71) in the frequency of high-intensity decelerations (*n* = 62) compared to accelerations (*n* = 35). This reflects positional-specific movement demands whereby wide midfielders are required to perform various changes in direction both before and after high-intensity efforts to meet technical and tactical requirements when in and out of possession [62, 63]. Additionally, coaches and other support staff should be aware of the fluctuations in high-intensity accelerations and decelerations that result from tactical changes in game play. For example, in the study by Tierney et al. [51], wide midfielders performed 20% more high-intensity decelerations when playing in a 3–4–3 (*n* = 66) compared to a 4–4–2 (*n* = 53) formation. These findings have important implications for the design of training micro-cycles. For instance, players who perform a high frequency of decelerations over a number of weeks may be at an increased risk of injury, whilst a moderate frequency may provide a protective effect, thereby reducing the chance of injury occurring [64].

Significant team and positional differences in the frequency of high-intensity decelerations compared to accelerations were also observed in all codes of rugby (*I*^2^ = 57–97%). Both rugby league and rugby union had a greater (SMD = 0.99 and 1.11, respectively) frequency of high-intensity decelerations compared to accelerations. In rugby sevens, the difference in high-intensity decelerations compared to accelerations was large (SMD = 1.56), although the CI overlapped both trivial positive and negative effects. Whilst rugby sevens is played under the same laws and pitch dimensions as rugby union, the fewer players per team (7 compared to 15) permits larger spaces [65], requiring players to possess exceptional acceleration and maximal speed capabilities to achieve success in both attacking (defenders beaten, line breaks) and defensive plays (defensive rucks, dominant tackles) [66]. Both studies [36, 49] included in our meta-analysis obtained estimates from international rugby sevens tournaments across multiple matches. In the study by Suarez-Arrones et al. [36], differences in the frequency of high-intensity accelerations and decelerations were trivial. However, the absolute number of high-intensity accelerations (*n* = 13.1) and decelerations (*n* = 13.6) per match was greater than for all positions reported in senior and under 20 international rugby union players [50], despite total game duration being up to 80% less than rugby union. Indeed, using a total game time of 14 min, this would represent an average density of high-intensity accelerations and decelerations of approximately 1 action per minute. This average density is different to that of Furlan et al. [49] who reported a very large (SMD = 3.07) difference in the number of high intensity decelerations (1.8 n·min) compared to accelerations (0.4 n·min) in international rugby sevens players.

Based on these studies, it seems that there could be a large variability in the frequency of high-intensity accelerations and decelerations required to be performed during international match play. This could be owing to a range of physical (high-speed running ability, resistance to muscle damage—especially on day 1 of tournaments, neuromuscular fatigue), technical (number of contacts, tackle proficiency), psychological (well-being, perceived recovery) and situational (tournament day, score during match, opposition world ranking, travel requirements) related factors that have been shown to influence the match-activity profiles of international rugby sevens players [67–73].

Nonetheless, such a high density of high-intensity accelerations and decelerations in combination with physical contacts (rucks, mauls, scrums, tackles) is likely associated with the significant increase in muscle damage [68, 72] that coincides with deficits in neuromuscular function [68] and psychological disturbances [72, 73] following rugby sevens match play. Because rugby sevens players are required to perform multiple matches on consecutive days, with little time (~ 3 hours) for regeneration, strategies that can help enhance and maintain players’ physical and psychological readiness between matches are essential to successful performance, and have been carefully considered in recent research [74, 75]. In fact, some of these practices may be transferable to those involved in the preparation of rugby league and rugby union players whose aim is to develop a higher intensity of game play, whilst simultaneously reducing the muscular damage commonly associated with these actions [72].

The higher frequency of high-intensity decelerations compared to accelerations in both rugby league and rugby union is likely associated with increased spatial constraints, which restrict opportunity for high-speed running, and thereby demands players to perform more rapid short deceleration movements [46]. Unique positional responsibilities associated with offensive and defensive actions imply that high-intensity decelerations will accrue through differing task demands. For example, forwards are involved in a heightened number of heavy collisions [41, 45] that demand deceleration prior to contact to successfully perform the skill and reduce the amount of load accumulated [76, 77]. In contrast, backs have less involvement in collisions and are further away from the ball, permitting opportunities to use rapid deceleration movements to perturb the defensive line [45, 50, 78].

Amongst the team sports included in this meta-analysis, Australian Football had the second largest difference (SMD = − 1.15) in the frequency of high-intensity decelerations (*n* = 51–125) compared to accelerations (*n* = 38–103). A larger pitch size than other team sports, coupled with a no ‘offside’ rule, permits a higher contribution of continued high-speed running [43]. Despite this, both high-intensity accelerations (~ 15%) and decelerations (~ 20%) have been shown to be the largest contributors to post-match markers of muscle damage, denoted by elevated levels of creatine kinase (CK) in Australian Football players [11]. Similarly, research by Young et al. [13] also found significant correlations between high-intensity accelerations and decelerations and CK levels in Australian Football players, but only the volume (represented by distance covered) of high-intensity decelerations was significantly different between the low- and high-CK groups. It is noteworthy that Australian Football players who cover more high-intensity deceleration distance also report a higher perceived match load, despite this being essential for increasing the amount of possessions and disposals of the ball that can contribute to match success [14]. This is exemplified by the match-activity profile of elite Australian Football players containing more high-intensity decelerations per minute than sub-elite players [30]. Collectively, these findings highlight the importance of high-intensity decelerations to Australian Football match-play performance together with the damaging consequences of these actions. Similar to recommendations previously suggested for soccer practitioners, those involved in the preparation of Australian Football players should look to implement interventions that reduce a player’s susceptibility to deceleration-induced tissue damage [11], likely arising from the intense eccentric muscle contractions experienced when braking abruptly.

In hockey, the results of our meta-analysis showed that only the defensive and midfield positions had a greater (SMD = 0.78) frequency of high-intensity decelerations (0.9–1.0 n·min) compared to accelerations (0.7–0.8 n·min). In the forward position, differences between high-intensity accelerations (0.9 n·min) and decelerations (1.0 n·min) were trivial, suggesting that some forward players may complete more high-intensity accelerations than decelerations during match play. Previous studies have also shown that forward positions accelerate to higher intensities more frequently than defenders and midfielders [79, 80]. This difference could be due to the unlimited interchange rule that reduces effective playing time; meaning less total distance and energy is expended, therefore allowing a higher intensity intermittent profile to be maintained [79]. From a tactical perspective, rotating forward positions more frequently can help to maintain high-intensity output across periods of play, resulting in more technical contributions and enhanced team performance statistics [81].

Although the frequencies of high-intensity accelerations have been shown to be different between positional roles in hockey, the relative frequency of high-intensity decelerations was similar across all positions (0.9–1.0 n·min). As the ability to decelerate at high intensity can also influence a player’s change of direction performance [82], these actions may have particular importance to hockey match-play performance outcomes. Furthermore, both high-intensity accelerations and decelerations have been shown to be the match-activity variables most sensitive to fatigue development during hockey match play [18]. Performance advantages could therefore be obtained by strategies that help to increase and maintain players’ capacity to both accelerate and decelerate rapidly throughout match play.

An interesting finding of the present meta-analysis was that all positional roles in American Football are required to perform more (SMD = 0.91–1.45) high-intensity accelerations (*n* = 15–38) compared to decelerations (*n* = 8–19) during competition. This finding was unique to American Football and supports the significant time and investment that is placed on the assessment and development of an American Footballer’s rapid acceleration and top speed capabilities. Indeed, these abilities have been shown to differentiate between drafted and non-drafted players in the National Football League (NFL) Scouting Combine [83] and are important in predicting future successful performance in the NFL, including the amount of prestigious accolades (i.e. Pro Bowl, All Pro) players achieve [84].

Despite the prevalence and clear importance of high-intensity accelerations to match performance in American Football, the lower frequency of high-intensity decelerations compared accelerations may have some very important implications. It is well known that to accelerate, rapidly high concentric leg extensor strength capacities are required [85, 86] to produce larger and more efficient horizontal ground reaction forces [87–92]. It has also been shown that habitual loading with a predominance of a concentric mechanical stimulus could result in muscle-tendon tissue properties that leave players more vulnerable to eccentric-induced dysfunction and injury risk [93, 94]. This vulnerability to eccentric load could be inevitable in American Football—up to 40% of the weekly player load arises from match play [95], with high-intensity sprinting activity constituting an important stimulus that can lead to neuromuscular adaptations associated with increased muscular power [96]. Research has also shown that despite NFL players having distinct anthropometric (height, mass) characteristics, players all accelerate in a similar manner relative to maximum velocity, and that this could be due to the homogenous sprint training programmes they complete in the 4- to 8-week period in preparation for the NFL 40-yard dash [97]. Consequently, NFL players capable of faster horizontal movement speeds will subsequently have greater braking demands [98], which if not accompanied by higher levels of eccentric strength will result in a worse change of direction ability [99] and an increased risk of injury occurring [100].

On this basis of these findings, practitioners supporting American Football players may need to prioritise loading strategies during training sessions that develop muscle-tendon tissue structures’ capacity to attenuate high eccentric forces. Practitioners should also be cognisant of periods during the season when American Football players may be more susceptible to eccentric-induced muscle damage, for example, following periods of training with a dominance of concentric conditioning in which vulnerability to damage can be increased (such as when preparing for the NFL 40-yard dash) [101].

### Frequency of Very High Intensity Accelerations Compared to Decelerations

Very high (> 3.5 m·s^−2^) intensity accelerations and decelerations were reported across four sports including American Football [42], rugby sevens [36], rugby union [50] and soccer [20]. The difference between very high intensity accelerations and decelerations in both American Football (SMD = 0.19) and rugby sevens (SMD = − 0.12) was trivial. However, the heterogeneity analysis showed a large (*I*^2^ = 92–94%) variation across the estimates (positions and teams) within each of these sports. In American Football, a noteworthy finding from the meta-analysis was that the offensive lineman was the only position reporting a greater frequency of very high intensity decelerations (*n* = 7) compared to accelerations (*n* = 2), with the difference being very large (SMD = − 2.47). The offensive linemen are required to operate in confined chaotic spaces around the scrimmage with a primary responsibility of blocking opponents from tackling their own team’s ball carrier [42]. The results of our meta-analysis suggest that these actions may rely heavily on high impulse braking actions that allow for rapid decelerations and directional changes to be made to manoeuvre effectively around such congested areas of the field, and in response to the dynamic unpredictable movements of their own and opposition players. The offensive linemen are also the heaviest of all positional roles, which may further augment the magnitude of braking forces required to decelerate such high whole body momentum. These factors contribute to a high-risk loading profile that increases the chances of soft-tissue injuries occurring within this specific positional role [102]. Practitioners should select specific exercises for offensive linesmen that target the development of the neuromuscular capabilities required to produce and attenuate the high forces associated with decelerating rapidly, whilst also ensuring a high level of perceptual-cognitive training that will harness the ability to skilfully apply braking forces during emergent and unpredictable situations. The evident unpredictability of loads associated with decelerating rapidly also have hugely important implications for the management of load throughout the season, and return to sports participation programmes following injury [103, 104].

Similar to the high-intensity category, soccer demonstrated the highest (SMD = − 3.19) frequency of very high intensity decelerations compared to accelerations. The SMD ranged from very large (− 2.40 to − 2.79) in defenders (*n* = 24 vs. 7) and attackers (*n* = 16 vs. 5) to extremely large (− 4.43) in midfielders (*n* = 32 vs. 8). Given the previously discussed consequences of high-intensity decelerations to match performance and the development of cumulative fatigue (during and post-match), additional research is needed to gain a more comprehensive understanding around the prevalence and significance of very high intensity decelerations to soccer match-play performance, and readiness to play.

### Temporal Changes in High and Very High Intensity Accelerations and Decelerations

Understanding how specific match-play activities may influence player fatigue and recovery profiles is of significant interest to practitioners [105]. The results of our meta-analysis show there was a small (SMD = 0.46–0.54) decrease in the frequency of high and very high intensity accelerations and decelerations from the first to the second half periods of match play. As higher intensity accelerations and decelerations have been particularly associated with post-match decrements in neuromuscular fatigue and perceptual disturbances, it is also likely that these actions have a particularly profound effect on changes to match-related movement ability and efficiency. Despite this consequence, limited studies have actually examined the actual fatigue response induced by actual match-play activities, but instead focused on using simulation protocols that induce a lower mechanical load that is reflected in lower levels of muscle damage (i.e. CK) and feelings of muscle soreness [105].

When examining the actual influence of specific match-play activities (soccer in this example), it has been shown that the distance and frequency of high-speed running completed by a player during match play can lead to decrements in the ability to produce horizontal forces when accelerating maximally [106]. This consequently leads to reductions in sprint performance times that could be decisive in critical match-play actions. The individual estimates obtained in our meta-analysis show that rugby sevens reported the largest decrease (SMD = 0.97) in the frequency of high-intensity accelerations from the first (*n* = 7.8) to the second half (*n* = 5.3) period of match play. Collectively, these findings suggest that a high frequency of accelerations together with the opportunity to sprint for longer distances (which is also apparent in rugby sevens match play) may be particularly detrimental to the ability to produce horizontal forces when accelerating at high intensity.

Despite these findings, to our knowledge, no previous study has examined the potential acute transient fatiguing effects of performing a high frequency of high-intensity decelerations during match play. Our meta-analysis shows that a small decrease in high and very high intensity decelerations occurs from the first to the second half periods of match play with the largest (SMD = 0.78) decrease (*n* = 67 to 56) reported in soccer. When rapid decelerations are required to be performed frequently following maximal sprint acceleration, fatigue and sprint performance are further exacerbated when compared to sprinting with no enforced deceleration [107]. Such a high frequency of rapid decelerations leaves players vulnerable to muscle damage, which can impair force production capacity leading to declines in the performance of activities such as sprinting and changing direction [108]. Future research should look to investigate the temporal changes in high-intensity accelerations and decelerations during match play, and the factors that could help to maintain repeated high-intensity acceleration and deceleration performance throughout match play.

### Methodological Limitations of Eligible Studies

Previous reviews examining the use of wearable GPS devices for quantifying match-activity demands have identified that there is a lack consistency and consensus in the methodological procedures used across studies [23, 25]. Using recent guidelines [34, 35], we produced a checklist to evaluate the methodological differences (data collection, data processing and normative profile) between studies in how high and very high intensity accelerations and decelerations were determined. To collect data, over 60% of the studies included in our meta-analysis used GPS devices (Catapult Sports; *n* = 2, STATSports; *n* = 4, GPSports; *n* = 3) with a 10-Hz sampling frequency. Using a 10-Hz sampling frequency, it has been shown that the occurrence of high-intensity accelerations and decelerations can be reliably obtained, although distance- and time-related variables are less reliable [109–111]. In studies that used a 5-Hz sampling frequency, the RoB was rated as high because this sampling frequency has been shown to be less reliable than 10 Hz [109, 110].

The MED and filtering technique used are two extremely important data processing features that can also significantly change the quality, reliability and usefulness of acceleration and deceleration data [34, 111]. The MED delineates the minimal time in which an acceleration or deceleration needs to be maintained above a pre-defined threshold for it to be identified as an effort. Even small changes (0.1 s) in MED can result in substantial differences in the frequency of high-intensity efforts [35], for example, a lower MED is capable of detecting shorter and higher rates of acceleration and deceleration, whilst also being more susceptible to measurement error that could result in multiple accelerations or decelerations being given to a single effort [35, 112]. To prevent this, criteria that delineate the end of an acceleration or deceleration could be used in conjunction with the MED, such as when the acceleration falls below 0 m·s^−2^ or a certain threshold [34, 35]. However, no study in our meta-analysis reported this information, meaning it cannot be discounted that the frequency of high-intensity accelerations or decelerations was overestimated. Furthermore, despite the importance of the MED, our checklist showed that only eight studies [17, 19, 20, 36, 43, 46–48] reported the MED, and across these studies the duration (0.2–1 s) selected was inconsistent.

Because low and high MEDs can result in over- and under-estimates, respectively, this again might raise doubt around the accuracy of the higher intensity acceleration and deceleration frequencies that are reported in current research studies. To aid comparisons between studies and to improve the accuracy of data reported, practitioners and researchers should consider carefully the criterion used to delineate both the start and end of an acceleration or deceleration, and also ensure this information is clearly reported within the methodology.

The data filtering technique used has also been shown to have a substantial influence on high-intensity acceleration and deceleration outputs [35, 111]. For example, large differences in acceleration and deceleration data can occur between and within manufacturers own proprietary software versions following updates, and when comparing manufacturer software-derived data to those obtained using independent raw processing methods [35, 111]. Eleven studies [10, 17–19, 36, 41, 42, 44, 45, 48, 50] included in our meta-analysis used manufacturers’ own proprietary software, five studies [16, 29, 43, 46, 49] used raw filtering methods, whilst three studies [20, 47, 51] did not provide any information on the filtering technique used. As the reliability and usefulness of high-intensity acceleration and deceleration data can be enhanced by careful consideration to the data processing technique used [35, 109, 111], future research should look to establish which acceleration and deceleration metrics and data processing methods provide the most valid, reliable and sensitive data outputs. With respect to this, an average acceleration-deceleration metric (Ave Acc/Dec), calculated by taking the absolute value of all raw acceleration and deceleration values then averaging them over the duration of a selected time period, has been found to have better reliability and sensitivity across a range of GPS devices than threshold-based approaches [109, 111]. Whilst this approach can provide an indication of the absolute acceleration and deceleration demands, it does not differentiate between different magnitudes of acceleration or deceleration. Similarly, it does not enable the identification of acceleration and deceleration density, and when acceleration and deceleration values are combined, it fails to differentiate the unique physiological and mechanical loading demands of these activities.

Finally, another methodological limitation was associated with the development of a ‘normative profile’. Based on previous research, we chose ten matches to be representative of a ‘normative profile’ [113]. However, we acknowledge future research is needed to specifically examine how many games are required to ascertain that the stabilisation of high-intensity accelerations and decelerations have occurred, notably with regard to inter-match variability. Nonetheless, using this criteria, five studies [10, 17, 19, 36, 49] were rated as a high risk of bias because of using fewer than ten matches and not presenting position-specific data.

### Limitations and Future Directions

Whilst the results of this meta-analysis have a number of evident limitations, a range of factors can be identified to help direct future practice aimed at measuring high-intensity accelerations and decelerations during match play. First, all studies included in the meta-analysis utilised ‘generic’ or ‘arbitrary’ high-intensity acceleration and deceleration thresholds. Although these ‘generic’ thresholds allow “like for like” comparisons, they do not take into account individual differences in maximal acceleration and deceleration capacities that can result in significant differences in data output, particularly at higher intensities, and in players with higher maximal accelerative capabilities [114]. Furthermore, Sonderegger et al. [115] showed that if the running speed immediately prior to an acceleration being initiated is not considered, a number of high-intensity accelerations could be missed. Whilst these few studies have made a contribution to enhancing the quantification of high-intensity accelerations, there is currently no research to date that has individualised thresholds based on a player’s maximal deceleration capacity. Because high-intensity decelerations permit the highest rates of velocity change, future research that adopts a threshold-based approach should look to establish exclusive high-intensity acceleration and deceleration thresholds, rather than using a shared threshold that is commonly adopted across current practice.

We also acknowledge that a major limitation of the threshold-based approach, together with other methods (i.e. Ave Acc/Dec) that have been proposed, is a lack of contextualisation with regard to the specific movement sequences and the technical and tactical requirements of different positional roles [63]. When additional layers of contextual information are provided, novel position-specific training interventions can be developed [116, 117]. Examples include the starting speed at which accelerations and decelerations are initiated, distance of the acceleration or deceleration, actions that precede or follow the acceleration or deceleration, and their technical or tactical purposes. Furthermore, because higher intensity accelerations and decelerations have been classified as the major external loads in team sports [5], further insights into the internal mechanical stresses placed on soft tissues during different magnitudes of acceleration and deceleration could be obtained by integrated inertial sensors providing estimates of foot impulses during accelerated or decelerated running using metrics such as a force load [112].

Finally, all studies included in this review reported average acceleration and deceleration data from players who completed at least 75% of match duration. Future research should look to analyse acceleration and deceleration occurrences across smaller time periods so the magnitude and temporal location of peak demands can be more precisely identified [109]. This approach could also be useful when analysing substitute players, whom upon entering the field of play may produce a higher frequency of intense accelerations and decelerations, therefore requiring different pre-entry warm-up strategies to ensure optimal preparation [118].

## Conclusions

High-intensity accelerations and decelerations are particularly important measures of external biomechanical load in team sports. This is the first meta-analysis to compare high and very high intensity acceleration and deceleration demands in elite team sports competitive match play. In all team sports, apart from American Football, there was a greater frequency of high and very high intensity decelerations compared to accelerations. There is a small reduction in the frequency of high and very high intensity accelerations and decelerations from the first to the second half periods of match play. These findings have important implications for practitioners involved in ensuring elite players are optimally prepared for the high-intensity biomechanical loading demands of competitive match play. This review has also highlighted that there is currently a lack of consensus or consistency in the methodological procedures used to quantify higher intensity accelerations and decelerations during match play when using GPS devices.

Future research should establish measurement procedures that allow for valid, reliable and precise information to be obtained on individual high-intensity acceleration and deceleration demands. Finally, to permit more accurate individualised programming prescription, other contextual information relating to how and when high-intensity accelerations and decelerations are occurring during match play, should also be provided.

## Electronic supplementary material

Below is the link to the electronic supplementary material.

Supplementary material 1 (DOCX 110 kb)
