# Supplementary material for: High-Intensity Acceleration and Deceleration Demands in Elite Team Sports Competitive Match Play: A Systematic Review and Meta-Analysis of Observational Studies
Source: Sports Med. 2019 Sep 10;49(12):1923–47. doi: 10.1007/s40279-019-01170-1 (PMC6851047; doi:10.1007/s40279-019-01170-1)
Supplement: Supplementary file 1 — Supplementary material 1 (DOCX 110 kb) [file 40279_2019_1170_MOESM1_ESM.docx]

Table S1. Model for classifying the validity of expert samples (modified from Swann et al. [33])

| **Variable** | **Score** | | | |  |
| --- | --- | --- | --- | --- | --- |
|  | **1** | **2** | **3** | **4** |  |
| **A. Competitive standard** | Compete in highest 4^th^ tier league in respective sport | Compete in highest 3^rd^ tier league in respective sport | Compete in highest 2^nd^ tier league in respective sport | Compete in top tier league in respective sport or International standard | **Within sport comparison** |
| **B. Professionalism of players/training schedule** | Not professional status, not full time training schedule | Semi-professional, part-time training schedule | Full time professional, full time training schedule | Full time professional, full time training schedule, international level players |  |
| **C. Competitive experience at the highest level** | Young players competing outside of the top tier (e.g. reserve, U23’s) | Young players competing in top tier (e.g., Premier League reserve, U23’s), senior players competing outside of top tier | Senior players competing in top tier of respective sport | Senior players competing in top tier of respective sport with international experience |  |
| **D. Competitiveness of sport in respective country** | Sport not in top 10; small sporting nation | Sport ranks 5-10 in country; small-medium sporting nation | Sport ranks in top 5 in country; medium to large sporting nation | National sport; large sporting nation | **Between sport comparison** |
| **E. Global competitiveness of sport** | Not Olympic sport; world-championships limited to a few countries; limited national TV audience | World championships limited to a few countries; national TV audience; limited international TV audience | Sport with regular international competition; semi-global TV audience | Sport with frequent major international competition; global TV audience |  |
| Formula for calculation of eliteness of population sample = [(A+B+C)/3)] x [(D+E)/2] | | | | | |

Table S2. Total score and classification of ‘eliteness’ (Swann et al. [33])

| Total | Classification |
| --- | --- |
| 1 - 4 | Semi-elite |
| 4.1 - 8 | Competitive elite |
| 8.1 - 12 | Successful elite |
| 12.1 - 16 | World class elite |

Table S3. Classifications of ‘eliteness’ given to each study sample (Swann et al. [33])

| **Study** |  | **Variable** | | | | |  | **Total, classification** |
| --- | --- | --- | --- | --- | --- | --- | --- | --- |
|  |  | **A** | **B** | **C** | **D** | **E** |  |  |
| Akenhead et al. [16] |  | 4 | 4 | 2 | 4 | 4 |  | 13.3,  World class elite |
| Coutts et al. [43] |  | 4 | 3 | 3 | 4 | 3 |  | 11.7,  Successful elite |
| Cummins et al. [29] |  | 4 | 4 | 4 | 4 | 3 |  | 14,  World class elite |
| Cunningham et al. [50] – U20 |  | 4 | 3 | 2 | 4 | 3 |  | 10.5,  Successful elite |
| Cunningham et al. [50] - Senior |  | 4 | 4 | 4 | 4 | 3 |  | 14,  World class elite |
| Dempsey et al. [45] |  | 4 | 4 | 4 | 4 | 3 |  | 14,  World class elite |
| De Hoyo et al [10] |  | 4 | 4 | 2 | 4 | 4 |  | 13.3 =  World class elite |
| Furlan et al. [49] |  | 4 | 4 | 4 | 3 | 3 |  | 12 =  Successful elite |
| Higham et al. [48] |  | 4 | 4 | 4 | 3 | 3 |  | 12 =  Successful elite |
| Johnston et al. [44] |  | 4 | 3 | 3 | 4 | 3 |  | 11.7 =  Successful elite |
| Jones et al. [41] |  | 4 | 4 | 4 | 4 | 3 |  | 14 =  World class elite |
| Kempton et al. [46] |  | 4 | 4 | 4 | 4 | 3 |  | 14 =  World class elite |
| Morencos et al. [18] |  | 4 | 2 | 3 | 3 | 1 |  | 6 =  Competitive elite |
| Oxendale et al. [47] |  | 4 | 4 | 4 | 4 | 3 |  | 14 =  World class elite |
| Russell et al. [17] |  | 4 | 4 | 2 | 4 | 4 |  | 13.3 =  World class elite |
| Russell et al. [19] |  | 4 | 4 | 2 | 4 | 4 |  | 13.3 =  World class elite |
| Suarez-Arrones et al. [36] |  | 4 | 4 | 4 | 3 | 3 |  | 12 =  Successful elite |
| Tierney et al. [51] |  | 2 | 3 | 2 | 4 | 4 |  | 9.3 =  Successful elite |
| Wehbe et al. [20] |  | 4 | 3 | 3 | 3 | 4 |  | 11.7 =  Successful elite |
| Wellman et al. [42] |  | 4 | 4 | 3 | 4 | 3 |  | 12.8 =  World class elite |
| *A* highest standard of performance, *B* success at the athletes highest level, *C* experience at the athletes highest level, *D* competitiveness of sport in athletes country, *E* global competitiveness of sport | | | | | | | | |

Table S4. Risk of bias in the measurement of outcomes (detection bias)

| **Study** | **A. Data collection** | | | | | | | | | **B. Data processing** | | | **C. Normative profile** | | |
| --- | --- | --- | --- | --- | --- | --- | --- | --- | --- | --- | --- | --- | --- | --- | --- |
|  | Software details provided | Sampling Frequency  > 10Hz | Same GPS unit for all players | Same GPS unit for all games | Preparation of units described | > 6 satellites obtained | HDP <1 | Reliability for ACC/DEC given/cited | **OVEERALL RATING** | Reported MED | Filtering technique explained | **OVEERALL RATING** | > 10 matches used | Reported position specific data | **OVEERALL RATING** |
| Akenhead et al. [16] | Y | Y | Y | Y | Y | Y | Y | Y | **+** | N | Y | **?** | Y | N | **?** |
| Coutts et al. [43] | Y | Y | Y | Y | N | NI | NI | Y | **?** | Y | Y | **+** | Y | Y | **+** |
| Cummins et al. [29] | N | N# | **N** | NI | N | NI | NI | **Y** | **-** | **N** | Y | **?** | NI | Y | **?** |
| Cunningham et al. [50] U20 | Y | Y | Y | Y | Y | N | NI | Y | **+** | N | Y | **?** | Y | Y | **+** |
| Cunningham et al. [50] Senior | Y | Y | Y | Y | Y | N | NI | Y | **+** | N | Y | **?** | N | Y | **?** |
| De Hoyo et al. [10] | Y | Y | **N** | NA | Y | NI | NI | **Y** | **?** | N | Y | **?** | N | N | **-** |
| Dempsey et al. [45] | Y | Y | **N** | NI | Y | NI | NI | Y | **?** | N | Y | **?** | N | Y | **?** |
| Furlan et al. [49] | N | N# | **N** | NI | N | NI | NI | N | **-** | N | Y | **?** | N | N | **-** |
| Higham et al. [48] | Y | N | **N** | NI | Y | NI | NI | N | **-** | Y | Y | **+** | Y | N | **?** |
| Johnston et al. [44] | Y | PY | N | Y | N | Y | Y | Y | **+** | N | Y | **?** | PY | Y | **+** |
| Jones et al. [41] | Y | Y | Y | NI | N | NI | NI | **Y** | **?** | N | Y | **?** | **Y** | N | **?** |
| Kempton et al. [46] | Y | N | Y | Y | N | Y | NI | Y | **?** | Y | **Y** | **+** | Y | Y | **+** |
| Morencos et al. [18] | Y | Y | **N** | NI | N | NI | NI | N | **-** | N | Y | **?** | Y | Y | **+** |
| Oxendale et al. [47] | **N** | Y | **N** | NI | Y | NI | NI | N | **-** | Y | N | **?** | N | Y | **?** |
| Russell et al. [19] | Y | Y | **Y** | NA | **Y** | NI | NI | N | **?** | **Y** | Y | **+** | N | N | **-** |
| Russell et al. [17] | Y | Y | Y | Y | Y | NI | NI | N | **?** | Y | Y | **+** | N | N | **-** |
| Suarez-Arrones et al. [36] | Y | N# | **N** | NI | Y | NI | NI | Y | **-** | Y | Y | **+** | N | N | **-** |
| Tierney et al. [51] | N | Y | **N** | NI | Y | NI | NI | Y | **-** | N | N | **-** | Y | Y | **+** |
| Wehbe et al. [20] | **N** | N | Y | Y | **Y** | N | NI | N | **-** | Y | N | **?** | N | Y | **?** |
| Wellman et al. [42] | Y | Y | **Y** | NI | Y | NI | NI | **Y** | **?** | N | Y | **?** | Y | Y | **+** |
| *Y* yes, firm evidence is available; *N* no, no evidence is available, *PY* probably yes, likely to be the case, *PN* probably no, not likely to be the case, *NA* not applicable, *NI* no information provided, ***(+)*** low risk of bias (plausible bias unlikely to seriously alter the results); (**?)** = unclear risk of bias (plausible bias that raises some doubt about the results); (**-)** = high risk of bias (plausible bias that seriously weakens confidence in the results); HDP = Horizontal dilution of precision; # = 15Hz GPS interpolated from 5Hz. | | | | | | | | | | | | | | | |

**Table S5.** Cochrane recommendations for interpretation of risk of bias within and across studies

| **Risk of bias** | **Interpretation** | **Within a study** | **Across studies** |
| --- | --- | --- | --- |
| Low risk of bias. | Plausible bias unlikely to seriously alter the results. | Low risk of bias for all key domains. | Most information is from studies at low risk of bias. |
| Unclear risk of bias. | Plausible bias that raises some doubt about the results. | Unclear risk of bias for one or more key domains. | Most information is from studies at low or unclear risk of bias. |
| High risk of bias. | Plausible bias that seriously weakens confidence in the results. | High risk of bias for one or more key domains. | The proportion of information from studies at high risk of bias is sufficient to affect the interpretation of results. |
